# Incidence and Management of Radial Nerve Palsies in Humeral Shaft Fractures: A Systematic Review

**DOI:** 10.7759/cureus.11490

**Published:** 2020-11-15

**Authors:** Erik M Hegeman, Michael Polmear, John P Scanaliato, Leon Nesti, John C Dunn

**Affiliations:** 1 Orthopaedic Surgery, Brooke Army Medical Center, San Antonio, USA; 2 Orthopaedics, William Beaumont Army Medical Center, El Paso, USA; 3 Orthopaedic Surgery, William Beaumont Army Medical Center, El Paso, USA; 4 Orthopaedic Surgery, Uniformed Services University of the Health Sciences, Bethesda, USA; 5 Orthopaedic Hand Surgery, William Beaumont Army Medical Center, El Paso, USA

**Keywords:** humerus fracture, radial nerve palsy, nerve exploration, radial nerve injury

## Abstract

Radial nerve palsies in closed humeral shaft fractures are common, with an incidence of 7%-17%. The management of radial nerve palsies in closed fractures is often expectant, with 70.7% spontaneously recovering within six months. A literature search was conducted for studies on radial nerve palsies in humeral shaft fractures from 2000-2018. A total of 4972 humeral shaft fractures were identified, with an incidence of 12.2% of primary radial nerve palsies. During the exploration, no neurological intervention was performed in nearly 41% of cases, and the most common finding was no evidence of any nerve lesion (35%). Those who underwent neurolysis were more likely to resolve when compared to primary repair or nerve grafting. Overall, there was a high rate of spontaneous radial nerve palsy recovery (85%) with radial nerve exploration increasing rates of resolution. While exploration demonstrates increased resolution, it is yet to be determined which fractures are indicated for nerve exploration.

## Introduction and background

The most recent systematic reviews performed on the incidence of and factors associated with radial nerve palsies in humeral shaft fractures were performed in 2013 and 2019 [1-2]. Mangan et al. concluded that the overall prevalence of radial nerve palsy was 12.3% in a population of 7,262 fractures [2]. Further, surgical exploration and fracture repair within three weeks of injury had a radial nerve recovery rate of 89.8%, which contrasted with 68.1% for those cases explored more than eight weeks from injury and 77.2% for those cases that were treated nonoperatively. In 2013, Li et al. determined the incidence of radial nerve palsies in these injuries to be 16.3% in a population of 1882 humeral shaft fractures [1]. In 2005, Shao et al. demonstrated an incidence of 11.8% in a population of 4517 fractures [3]. Humeral shaft fractures represent 1%-3% of all reported fractures [4]. The high incidence of radial nerve palsy in humeral shaft fractures makes this the most common peripheral nerve injury in long bone fractures [5].

Factors associated with persistent radial nerve palsies have yet to be fully elucidated due to the heterogeneous nature of reporting epidemiological data. Factors that have been previously described to be associated with radial nerve palsies are the location of the fracture, fracture pattern, mechanism of injury, the energy of the underlying fracture etiology, and type of surgical fixation [4]. Current literature shows that middle and distal third diaphyseal transverse and spiral fractures have a statistically higher incidence of primary radial nerve palsies [3]. In addition, fractures caused by higher energy mechanisms of injury such as motor vehicle accidents (MVAs), gunshot wounds (GSWs), and direct impacts are more likely to cause radial nerve palsies than lower energy mechanisms [6]. In a meta-analysis performed by Zhao et al. in 2017, iatrogenic radial nerve palsy was seen more often when open reduction plate fixation (ORPF) was performed compared to minimally invasive plate osteosynthesis (MIPO) and intramedullary nailing (IMN) [7].

Overall, there is a high rate of spontaneous recovery in radial nerve palsy. The rate of recovery of radial nerve function following radial nerve palsy when treated with prolonged observation is as high as 71%, with a mean follow-up duration of 30.1 months (5.5-80) [4]. The rate of recovery seen with exploration and nerve interventions increases the overall rate of recovery to 88.1%, with a mean follow-up time of 30.1 months (5.5-80) [4]. While the majority of studies support expectant management for all radial nerve palsies that occur in closed humeral shaft fractures, there are proponents who advocate for early surgical exploration [2,7-10].

The purpose of this study is to characterize the incidence of radial nerve palsies in both humeral shaft fractures treated operatively and non-operatively, as well as to further characterize prognostic factors for nerve recovery and incidence of nerve lesions at the time of exploration. Our hypothesis is that higher energy mechanisms of injury in diaphyseal humerus fractures will have the lowest rate of radial nerve palsy recovery and neuropraxia will be the most common finding at the time of exploration when accounting for all RNPs.

## Review

Materials and methods

Search Strategy

A computerized search was conducted of PubMed, EMBASE, and the Cochrane databases in February 2019, according to the Preferred Reporting Items for Systematic Reviews and Meta-Analyses (PRISMA) model for randomized controlled trials and for prospective and retrospective studies on radial nerve palsies in humeral shaft fractures. The search strategy was created using the help of a research librarian and used a combination of keywords, including “radial nerve palsy”, “radial nerve injury”, “radial nerve lesion”, “radial nerve”, “humerus”, “humeral shaft”, “humeral diaphysis, “humerus fracture”, “humeral shaft fracture”, “fracture”.

Criteria for Eligibility

Exclusion criteria were as follows: (1) duplicate studies or case reports, (2) pediatric populations defined as studies investigating patients less than 16 years old, (3) pathologic fractures or periprosthetic fractures, (4) publication date prior to 2000, and (5) non-English language.

Selection of Studies

Studies were screened independently by a single reviewer using the search strategy and exclusion criteria described above. Clearly irrelevant studies were discarded. Uncertainty on whether to include studies for further analysis was determined by review by two additional authors.

Extraction of Data

Relevant information regarding fracture location, type of fracture using Arbeitsgemeinschaft für Osteosynthesefragen (AO) classification, operative and non-operative fracture fixation, method of fixation, mechanism of injury, development of primary and secondary radial nerve palsy, time to palsy resolution, and nerve findings at exploration were extracted by the primary author (Hegeman). The data extracted were reviewed for completeness and accuracy (Polmear and Scanaliato). Primary radial nerve palsy was assumed from the time of injury to the time of initial reduction or fixation. Radial nerve palsies that occurred after reduction or fixation attempts were made were categorized as secondary radial nerve palsies. At the time of exploration, nerves described as ‘intact’ or ‘contused’ were grouped if no intervention was performed. Spontaneous nerve recovery is defined in this study as a return to 4/5 motor strength in the radial nerve distribution, with minimal to negligible paresthesias without exploration and nerve interventions (neurolysis, repair, or graft) at 12 months of clinical follow-up. Early exploration is defined as prior to six weeks from initial injury; explorations performed after this were categorized as late explorations. High-energy mechanisms of injury included low and high-velocity gunshot wounds (GSWs), motor vehicle accidents (MVAs), falls from heights greater than 10 feet, and severe direct impacts. Low-energy mechanisms of injury included falls from standing and low height falls less than 10 feet.

Statistical Analysis

Demographic, surgical, and outcome data were recorded for each study. Dichotomous data were compared using odds ratios (OR) and 95% confidence intervals (CI). For continuous variables, mean differences, standard deviations, and 95% confidence intervals were calculated. Statistical significance was defined as an alpha (p) ≤ 0.05.

Results

Literature Search

**Figure 1:**
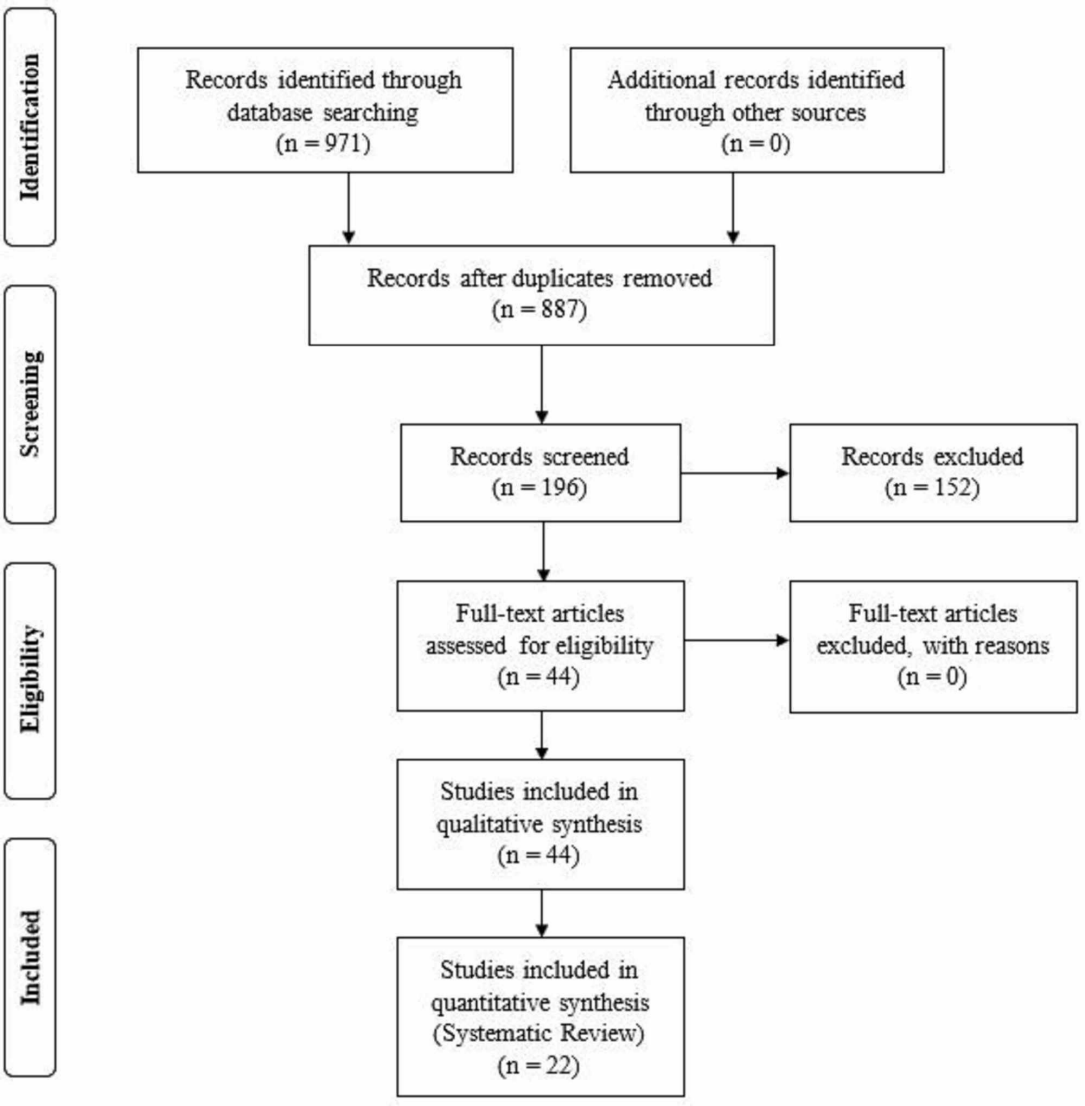
Literature and Study Selection

A comprehensive search of the PubMed, EMBASE, and Cochrane databases retrieved 971 articles. We excluded 84 duplicates and 691 unrelated articles. The remaining 196 abstracts were reviewed for inclusion and exclusion criteria, and 44 articles were examined in full. Of these, 22 articles were excluded and not used in qualitative analysis. The remaining 22 articles were included and reviewed systematically (Figure 1).

Characteristics of Studies

Seventeen studies provided data on humeral shaft fractures in their population [3,5,7,9,11-24]. Five studies provided information regarding findings at exploration but did not provide background epidemiological fracture data [8,10,22,25-26]. Overall, 4,972 fractures were identified in 4,969 patients with three cases of bilateral humerus fractures. Twelve studies offered conclusions regarding exploration in primary and secondary radial nerve palsies. None of the studies identified supported early exploration for primary radial nerve palsy, defined as within six weeks of injury, for closed primary radial nerve palsies in simple fracture patterns (12A). One study recommended early exploration for wedge and spiral mid-shaft fractures (12B and 12C) [24]. Two studies recommended early exploration for secondary radial nerve palsies [7,22]. Three studies favored early exploration for fractures undergoing ORIF [8-9,23]. Four studies advocated early exploration in high-energy mechanisms of injury with a concomitant primary radial nerve palsy [9,22-23,26].

Demographics

**Figure 2:**
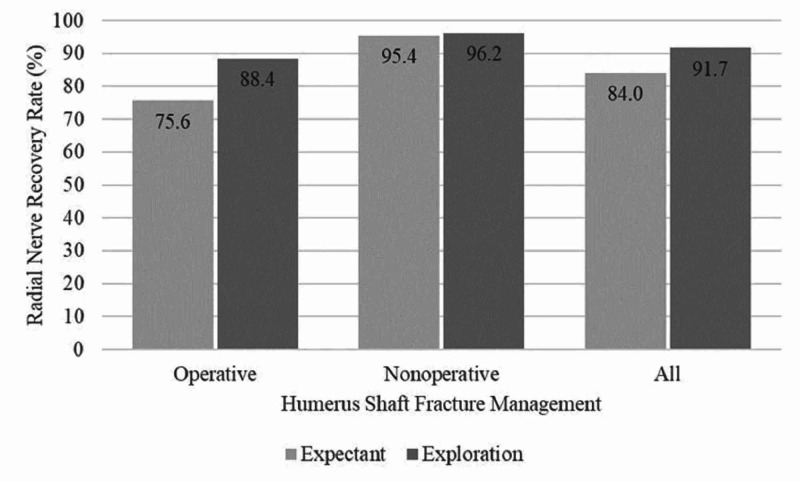
Radial Nerve Recovery Rate in Operative, Non-Operative, and all Humeral Shaft Fractures With and Without Nerve Exploration

Thirteen studies reported age with an average of 44.8±14.4 years (Table 1) [4,6-8,12-14,16,18,20,24-25]. Seventeen studies identified a total of 4,972 humeral shaft fractures of which there were 607 primary radial nerve palsies; giving an overall incidence of 12.2%. The distinction of open and closed fracture patterns was described in 11 studies, totaling 2,396 fractures; of these 10.5% (255 of 2396) were open fractures and 89.5% (2141 of 2396) were closed fractures. There was no statistical difference between the presence of radial nerve palsies when comparing open to closed fractures (OR 0.89, 95% CI 0.56-1.46, p=0.094). Only three studies with 1,290 fractures (25.9% of 4972) reported on anatomic location of the humeral shaft fracture associated with radial nerve palsies. In total, 303 (23.4%) fractures were described as proximal-, 624 (48.4%) middle-third, and 363 (28.1%) as distal-third. Six studies reporting on 1912 (38% of 4972) fractures provided data regarding fracture pattern; 833 were described as 12A (43.6%), 548 as 12B (28.7%) and 531 as 12C (27.8%). The overall rate of radial nerve palsy recovery, which included spontaneous resolution and nerve interventions at the time of exploration in both the operative and non-operative groups, was seen in 85.8% (521 of 607) of radial nerve palsies with a mean follow-up of 48 months. Radial nerve palsy resolution was more likely in those who underwent exploration than those who only received expectant management (OR 1.74, 95 CI 1.27-2.39, p=0.0006) (Figure 2).

**Table 1:** Study Demographics NR: Not reported

Author	Mean Age (Years)	Humeral Shaft Fractures	Operative		Nonoperative		Primary RNP		Energy Type		Fracture Type		Follow-Up (Years)
									High	Low	Open	Closed	
Ekholm [4]	56	361	283	78%	78	22%	33	9%	79	322	8	353	8
Venouziou [6]	39	48	48	100%	NR	0%	18	38%	NR	NR	9	39	5.5
Lang [7]	47	615	615	100%	NR	0%	55	9%	NR	NR	NR	NR	1
Schwab [8]	49	151	151	100%	NR	0%	20	13%	NR	NR	5	146	1.2
Nachef [10]	NR	373	373	100%	NR	0%	43	12%	NR	NR	NR	NR	1.5
Claessen [12]	45	325	325	100%	NR	0%	66	20%	190	135	47	278	NR
Mahabier [13]	61	186	95	51%	91	49%	13	7%	32	154	0	186	NR
Westrick [14]	33	296	227	77%	69	23%	87	29%	NR	NR	99	197	NR
Wang [15]	NR	707	707	100%	NR	0%	NR	0%	NR	NR	NR	NR	5.5
Denard [16]	36	213	150	70%	63	30%	NR	0%	NR	NR	2	212	NR
Bumbasirevic [18]	39	530	16	3%	514	97%	117	22%	NR	NR	30	500	NR
Pailhe [19]	51	225	225	100%	NR	0%	20	9%	NR	NR	NR	NR	9.5
Grouse [20]	43	85	85	100%	NR	0%	19	22%	NR	NR	0	85	NR
Duygun [21]	NR	24	24	100%	NR	0%	NR	0%	NR	NR	NR	NR	2
Grass [22]	NR	38	38	100%	NR	0%	15	39%	NR	NR	NR	NR	2.1
Belayneh [24]	49	175	77	44%	98	5%	25	14%	NR	NR	1	174	1
Sarmiento [25]	36	620	NR	0%	620	100%	76	12%	378	242	155	465	8.6
Total or Weighted Mean	44	4972	3439	69%	1533	31%	607	12%	679	853	356	2635	3.3

Operatively Treated Fractures

Overall, 69.1% (3,439 of 4,972) fractures underwent operative fixation. The method of fixation was described in 13 studies and 1,550 fractures. Open reduction internal fixation (ORIF) was the most common fixation method utilized occurring in 79.5% (1,227 of 1,550) fractures described, followed by intramedullary nailing (IMN) in 19.0% (303 of 1,550) and external fixation (ex-fix) in 1.3% (20 of 1,550) of the described fractures. Primary radial nerve palsy occurred in 329 operatively treated fractures, accounting for 54.2% (329 of 607) of primary radial nerve palsies included in this review. The rate of primary radial nerve palsy in the operative group was 9.5% (329 of 3439 fractures). The rate of spontaneous radial nerve palsy recovery in this group was 75.6% (249 of 329 radial nerve palsies) (Figure 2). Operatively treated fractures were less likely to have primary radial nerve palsies on presentation as compared to non-operatively treated fractures (OR 0.56, 95% CI 0.47-0.67, p<0.0001). The overall rate of radial nerve palsy recovery in the operative group, which included spontaneous recovery and those palsies that recovered with exploration and nerve interventions was 88.4% (291 of 329 radial nerve palsies). Statistically, those radial nerve palsies that underwent exploration and nerve intervention were more likely to resolve than those radial nerve palsies treated expectantly (OR 2.46 95% 1.61-3.75 p<0.0001). Secondary radial nerve palsies in the operative group were seen in 4.7% (165 of 3,439) patients. The rate of spontaneous recovery in secondary radial nerve palsies occurred in 96% of patients (159/165), the additional six patients underwent exploration, with no functional recovery seen in an average of 44 months of follow-up and underwent salvage therapy with tendon transfers.

Non-Operatively Treated Fractures

Overall, 30.8% (1,533 of 4,972) fractures underwent non-operative management with splinting or bracing, accounting for 30.8% of all fractures identified (1,533 of 4,972) [4,13-14,16,18,24-25]. In the non-operative group, there were 241 primary radial nerve palsies identified, giving an incidence of 15.7% (241 of 1,533) in this group. Spontaneous radial nerve palsy recovery in this group occurred in 95.4% (230 of 241) of radial nerve palsies (Figure 2). The remaining 11 patients with persistent radial nerve palsies underwent late explorations of which two fully recovered and the other nine had incomplete or failed recoveries. Radial nerve palsy recovery was statistically more likely to occur in the non-operatively treated humeral shaft fractures as compared to the operative group (OR 6.71 95% CI 3.48-12.93 p<0.0001) while persistent radial nerve palsies that failed expectant management were more likely to occur in the operative group (OR 2.73 95% CI 1.36-5.45 p=0.004).

Mechanism of Injury

Eleven studies reported on the mechanism of injury accounting for 30.8% of fractures identified (1,532 of 4,972). In total, 44.3% (679 of 1532) of identified fractures were classified as having a high and 55.6% (853 of 1,532) were described as occurring from a low-energy mechanism of injury. The most common injuries in the high-energy group were MVAs and GSWs while the most common injury in the low-energy group was fall from standing height. Primary radial nerve palsies were statically more likely to occur in the low-energy group as compared to the high-energy group (OR 1.41 95% CI 1.11-1.79 p=0.0043). Additionally, the low-energy radial nerve palsy group was more likely to have a resolution of radial nerve palsy as compared to the high-energy group (OR 2.83 95% CI 1.46-5.48 p=0.002). There was no statistical difference seen in the distribution of high and low-energy groups when compared in the operative and non-operative groups respectively (OR 1.23 95% CI 0.94-1.65 p=0.11).

Incidence of Nerve Lesions and Interventions Performed at Exploration

**Figure 3:**
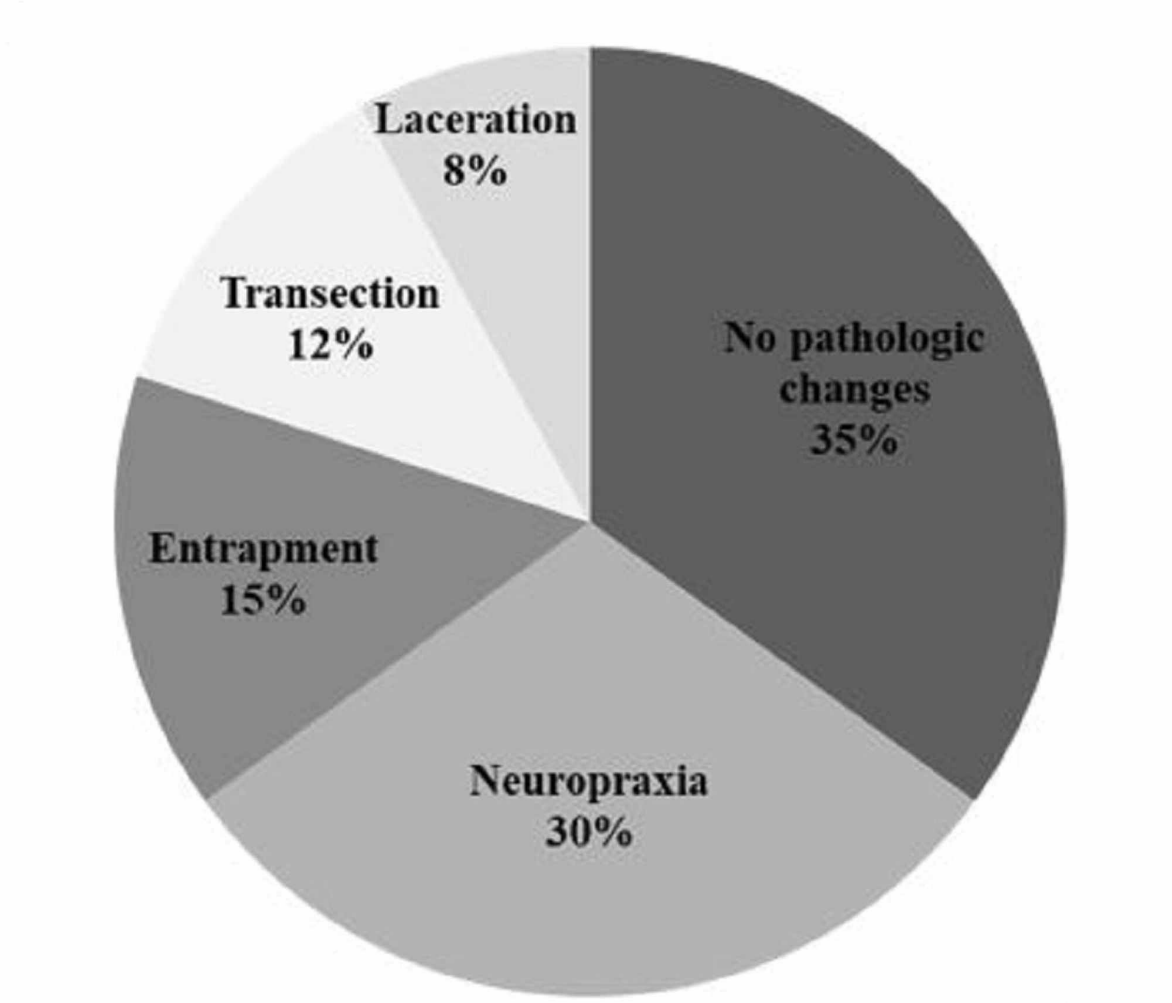
Radial Nerve Findings at Time of Exploration in 200 Cases

Radial nerve exploration was described in 22 studies, which included five studies that weren’t included in the epidemiological analysis due to insufficient reporting [9,11,23-25]. Exploration was performed in 47.1% of identified radial nerve palsies in this population (340 of 722). Early exploration was described in 90.5% of exploration cases (309 of 340) and exclusively occurred in the operatively treated fractures group. There were 12 described late explorations in the operative group and 19 described in the nonoperative group giving an incidence of 9.5% (31 of 340) in this group. Although 340 explorations were performed, only 200 provided useful data to further characterize findings at exploration and outcomes. The most common finding during exploration was an intact nerve with no evidence of pathologic changes that occurred in 35% (70 of 200) explorations (Figure 3). The resolution of radial nerve palsy was observed in 97.1% (68 of the 70) of the described intact nerves with no visible nerve lesion. The next most common finding at exploration was evidence of neuropraxia or scar tissue formation, which was described in 30% (60 of 200) explorations. Of the 60 cases of neuropraxia at the time of exploration, 96.6% (58 of 60) resolved. Entrapment occurred in 14.5% (29 of 200) explorations with 100% (29 of 29) entrapments resolving with nerve extraction at the time of early exploration within six weeks. Axonotmesis occurred in 8.5% (17 of 200) explorations with 64.7% (11 of 17) radial nerve palsies resolving with neurolysis and/or direct repair. Neurotmesis occurred in 12% (24 of 200) explorations with 9% (2 of 22) leading to full resolution of radial nerve palsy with direct repair ± grafting.

**Figure 4:**
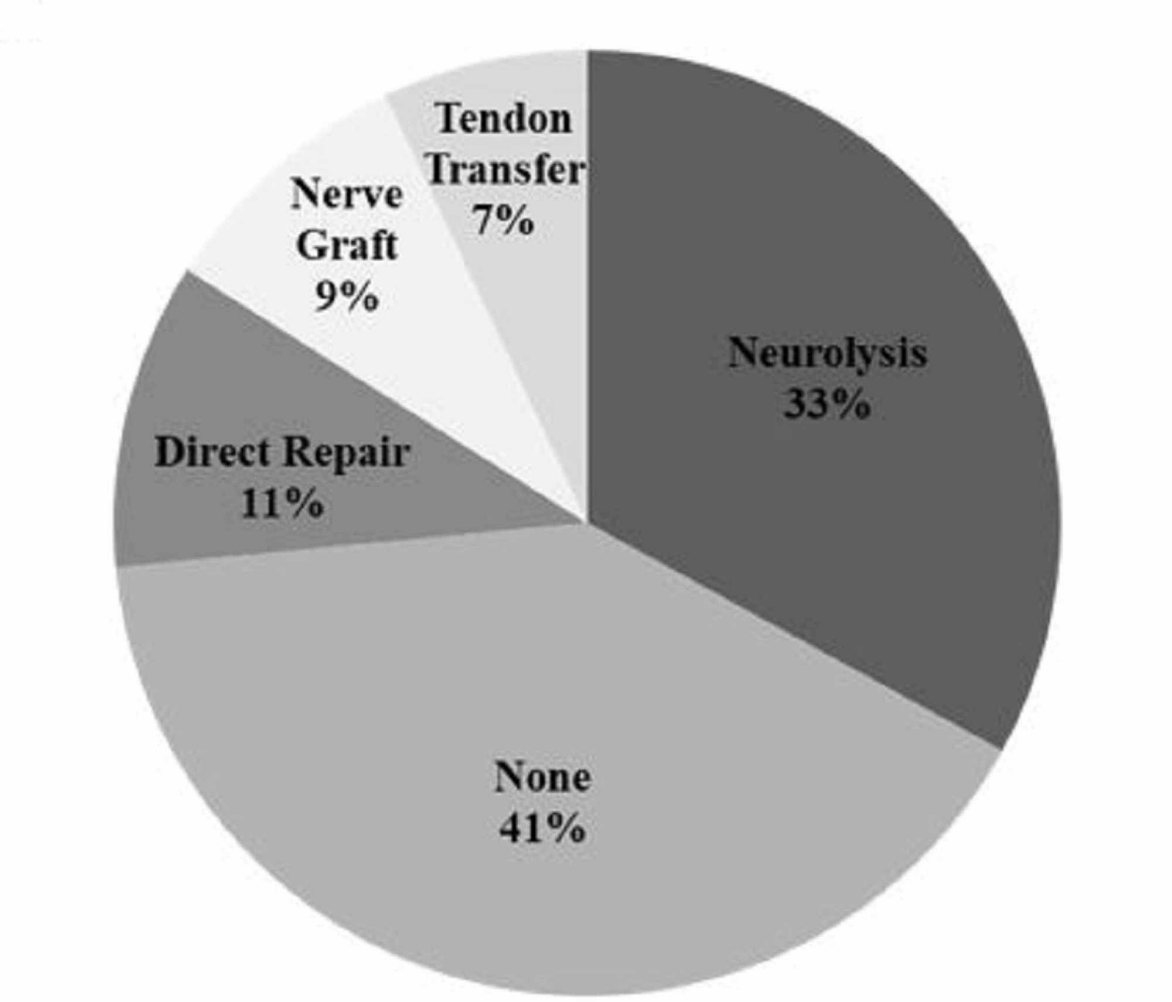
Radial Nerve Interventions at the Time of Exploration in 200 Cases

A portion, 40.5% (81 of 200), of explorations did not undergo any nerve interventions at the time of exploration. For those cases with intervention, the most common was neurolysis occurring in 55.4% (66 of 119), followed by direct repair 17.6% (21 of 119), then by nerve grafting 15.1% (18 of 119) (Figure 4). Tendon transfer occurred in 11.9% of interventions (14 of 119) performed, which were used as a salvage procedure due to persistent radial nerve deficit. The odds of recovering from radial nerve palsy were dependent on the method of nerve intervention performed. Neurolysis was most likely to recover when compared to direct repair (OR 9.27 95% CI 2.9-29.5 p=0.0002) and nerve grafting (OR 5.36 95% CI 1.56-18.34 p=0.007). However, when comparing direct repair and nerve grafting, no statistical difference was observed in terms of the difference in radial nerve palsy recovery (OR 0.57 95% CI 0.16-2.07 p=0.4). The overall rate of tendon transfers performed was 1.9% (14/722) for radial nerve palsies.

Mean Time to Recovery

In total, nine studies reported time to full radial nerve palsy resolution, which averaged to 23.6±5.9 weeks (Table 2) [6-7,9,11,15,19,23-24,27].

**Table 2:** Time to Radial Nerve Palsy Recovery in Operatively Treated Humeral Shaft Fractures

Study	Humeral Shaft Fractures	Mean (weeks)	Standard Deviation (weeks)
Venouziou 2011 [6]	39	20	4
Lang 2017 [7]	47	26.7	8.9
Han 2017 [9]	15	20	8
Yorukoglu 2016 [11]	24	12	4
Wang 2009 [15]	707	16	6.6
Pailhe 2015 [19]	51	60	6
Korompilias 2013 [23]	12	21.6	1
Belayneh 2019 [24]	49	19.1	10
Ring 2004 [27]	14	24	6
Total or Weighted Mean	958	19.3	6.6

Discussion

Demographics

This review identified 607 primary radial nerve palsies in 4,972 fractures giving an incidence of 12.2%, which is similar to a previous review by Shao in 2005 (11.8%) and a new 2019 review by Mangan et al. (12.3%) [2,3]. The overall rate of resolution determined by this study of 85.8% is consistent with findings by Shao et al. in 2005 of 88. [3].

Radial Nerve palsy in Operative and Non-Operative Treated Fractures

The rates of primary radial nerve palsy are higher in non-operatively (15.7%) compared to operatively treated fractures (9.5%, p=0.004). Functional bracing was the most common form of non-operative management in closed fractures, which comprised the majority of fractures included in this study, and secondary bone healing in the absence of absolute or relative stability may be irritative to the radial nerve [28]. High-energy closed fractures should be monitored more closely than low-energy closed fractures because statistically high-energy radial nerve palsies are less likely to resolve (p=0.001) when compared to low-energy mechanisms. Secondary radial nerve palsies encountered in the operative group had a high rate of recovery (96%), which suggests that secondary radial nerve palsies after ORIF should not be a definitive indication for exploration.

The high rate of primary radial nerve palsy resolution in the non-operative group of 96.3% supports current management algorithms that support a conservative approach to primary radial nerve palsies in this group. The average wait time for nerve recovery has been described in the literature as between three and six months [1]. In operatively treated humeral shaft fractures complicated by radial nerve palsies, the rate of spontaneous radial nerve palsy recovery is much lower at 75.6%, which increased dramatically to 88.4% with interventions performed on the radial nerve (p=<0.0001). In this population, radial nerve palsy management varies by surgical approach with some authors preferring a posterior approach to visualize the radial nerve at the risk of traction injury with retractors in contrast to the anterior and anterolateral approaches that do not visualize the radial nerve. Using the extent of radial nerve palsy on presentation, fracture location, fracture pattern, planned management, and mechanism of injury are important variables in determining the potential benefit of radial nerve exploration.

Mechanism of Injury

The findings of primary radial nerve palsies occurring more often in the low-energy mechanism of injury is not consistent with studies by Venoziou et al. and Ring et al. who stated that primary radial nerve palsies were more likely to occur in the high-energy mechanism of injury groups [5,26]. A possible explanation may lie in the direction of the force vector applied to the humerus at the time of injury. In higher energy mechanisms, such as MVAs or GSWs, it possible that the force applied to the humerus may be directed from a more anterior (anterolateral) to posterior (posteromedial) direction while the lower energy mechanism such as a fall from standing may direct forces from a posterior (posteromedial) to an anterior (anterolateral) direction. Anatomically, as the radial nerve travels in the spiral groove and through the lateral intermuscular septum, it lies closest to the posterior cortex of the humerus, which in a posterior to anterior force vector may be compressed against the posterior cortex of the humerus or stretched at the relatively rigid connection at the lateral intermuscular septum [29-30].

Incidence of Nerve Lesions and Interventions Performed at Exploration

Our study demonstrated that 27% (200 of 722) of primary radial nerve palsies underwent exploration. In 40.5% of these cases, no interventions were performed on the nerve. This supports that early exploration for primary radial nerve palsies even with high-energy mechanisms may not clearly identify nerve pathology and should be a component of pre-operative counseling. Conversely, evidence of neuropraxia was observed in 30% of cases and most often treated with neurolysis. Further, entrapment (15%), transection (12%), and laceration (8%) were found on exploration and benefited from intervention, accounting for the high rate of radial nerve palsy resolution in explored versus unexplored radial nerves. Mangan et al. arrived at a similar conclusion and advocated for early intervention to improve outcomes in these cases with higher grade nerve injuries [2].

Limitations

This study was limited by the heterogeneous nature of reporting demographics and inherent bias in reviewing retrospective data. All included studies were retrospective cohorts and series that reported on radial nerve palsies in operatively (69%) more often than non-operatively (31%) treated fractures. Further, there was insufficient reporting to control for other important variables that affect nerve recovery such as surgical approach, type of fixation, and rehabilitation course.

## Conclusions

The incidence of primary radial nerve palsy in this population of 4,972 humeral shaft fractures was 12.2%, which is consistent with previous studies. The rates of spontaneous resolution in the operative (75.6%) and non-operative (96.3%) groups had a combined rate of 85.8%. Radial nerve palsies were encountered less often in operatively (9.5%) than non-operatively (15.7%) treated humeral shaft fractures (OR 0.56 95% 0.47-0.67 p<0.0001). High-energy mechanisms of injury were less likely to cause radial nerve palsies when compared to low-energy groups (OR 0.37 95% 0.29-0.49 p<0.0001); however, high-energy injuries were also less likely to resolve (OR 0.35 95% 0.18-0.68 p=.002). Fractures treated operatively were more likely to undergo exploration compared to the non-operative group (OR 6.71 95% 1.61-3.75 p<0.0001). The rate of full neurologic recovery in the operative group without exploration was 75.6%, which increased to 88.4% with early or late explorations. Given these findings early exploration of all RNPs is not supported, although early exploration to identify nerve entrapment, laceration and transection may lead to improved outcomes. Further studies are needed to identify which specific fracture characteristics (such as degree of displacement and pattern and level of injury) are associated with nerve injuries that can be addressed with early exploration.
